# Developing multi-target therapeutics to fine-tune the evolutionary dynamics of the cancer ecosystem

**DOI:** 10.3389/fphar.2015.00209

**Published:** 2015-09-24

**Authors:** Lei Xie, Philip E. Bourne

**Affiliations:** ^1^Department of Computer Science, Hunter College, The City University of New YorkNew York, NY, USA; ^2^The Graduate Center, The City University of New YorkNew York, NY, USA; ^3^Office of the Director, National Institutes of HealthBethesda, MD, USA

**Keywords:** multi-target drug, cancer evolution, non-linear dynamic system, polypharmacology, cell-cell communication

Multi-target therapies, either in combination or in sequential order, have been advocated to combat intrinsic and acquired resistance to anti-cancer drugs (Holohan et al., 2013; Yardley, 2013). However, the effectiveness of multi-target anti-cancer therapy in the clinic is limited. The selection of cancer cells obeys Darwin's law of evolution. Under the pressure of drug perturbation, the cancer cell can adapt versatile molecular and cellular mechanisms for survival, and often evolves into more aggressive or metastasis phenotypes (Holohan et al., 2013). At the molecular level, acquired mutations resulting from drug treatment may modify drug metabolism (e.g., increasing efflux, decreasing uptake, and enhancing detoxification etc.) and alter drug-target interactions. At the cellular level, multiple pathways support the survival of cancer cells. The inhibition of one pathway may result in the activation of an alternative pathway. Although novel approaches to optimizing combination therapies have been proposed to defer these drug resistance mechanisms (Crystal et al., 2014; Wang et al., 2015), intra-tumor heterogeneity that have been observed ubiquitously may make the drug combination fail (McGranahan and Swanton, 2015). Polygenic drug-resistance mechanisms are present in sub-clones prior to the initiation of therapy (Bozic et al., 2013). If the therapy cannot target all sub-clones that drive the cancer progress in a fast-killing mode, it would prompt the rapid growth of sub-clones that are not sensitive to the treatment (Gatenby et al., 2009). Unfortunately, the number of driver mutations in advanced tumors is substantial (Gerlinger et al., 2014). It could be an impossible mission to target all driver mutations. The existence of cancer stem cells adds another dimension of complexity. The conventional single or combinational anti-cancer drug is incapable of killing cancer stem cells. When a cancer cell is killed by chemotherapy, it could send signals to stimulate the proliferation of the cancer stem cell, leading to the repopulation of the drug-resistance tumor (Kurtova et al., 2015). Thus, new strategies are needed to combat anti-cancer drug resistance with the goal to improve the effectiveness of anti-cancer therapy.

Cancer cells originate from the host's normal cells, but eventually turn into a new “pathogen” species. To eliminate anti-cancer drug resistance, we may borrow an idea from anti-bacterial drug discovery. Anti-virulence has emerged as a novel concept in addressing the challenge of antibiotic resistance (Rasko and Sperandio, 2010). Instead of killing bacteria, the anti-virulence drug interferes with the bacterial virulence and/or cell-cell communication, disrupts pathogen-human interactions, or enhances the host's inner immunity. The rationale is that the bacterium is less likely to evolve into a drug-resistant strain when facing less evolutionary pressure. As the cancer adapts similar mechanisms to the bacterium in acquiring drug resistance, the “anti-virulence” strategy could be applied as an anti-cancer therapy. Recent successes in anti-cancer immune therapy open a new door to exploring the “anti-virulence” strategy in cancer treatment (Johnson et al., 2015). If the cancer can be controlled as a less aggressive or non-metastasis type, it may be possible to cure the cancer by boosting anticancer immunity (Carmi et al., 2015). On the contrary, the chemotherapy may stimulate the production of immunosuppressive molecules (Shalapour et al., 2015). As a result, the patient's anti-cancer immune response is inhibited. Along these lines, adaptive therapy has been proposed to control the tumor growth by permitting the survival of drug sensitive cells. In this way, the growth of drug-resistant clones could be surpassed (Gatenby et al., 2009). In some cases, the cancer can be treated as a chronic disease when transferring the cancer cell into a quiescent state (Aguirre-Ghiso, 2006). For example, chronic myeloid leukemia (CML), chronic lymphocytic leukemia (CLL), and low grade non-Hodgkin's lymphoma (NHL) are slow-growing cancers. Patients can live with them for many years.

The various heterogeneous types of cancer cells form an ecosystem, cooperating and competing with each other for nutrients and spaces from the harsh environment. For example, sustained angiogenesis, one of the hallmarks of cancer, relies on the cooperation of co-existing cell lineages (Floor et al., 2012). The cooperating sub-clones either bear complementary traits or play a different role of producer or consumer of “public goods” such as diffusible growth factors (Korolev et al., 2014). Based on theoretical, experimental, and clinical results of ecology, microbiology, and cancer research, it has been proposed that tuning the population dynamics of cancer cells can be a powerful strategy in developing an anti-cancer therapy (Korolev et al., 2014). By either changing the tumor microenvironment, or confusing cancer cell-cell communications, the whole cancer ecosystem can be controlled, even eliminated.

To determine the evolutionary dynamics of the cancer ecosystem, and the drug targets that can modulate its evolutionary trajectory, we need a deep understanding of not only the drug response and resulting evolution of individual cell types but also the emergent properties of the whole system under a diverse genetic and environmental background, which is more than a simple summarization of the behavior of all cells. Recent advances in cancer biology, single cell technologies, next-generation sequencing, and systems biology provide great opportunities to dissect the evolution of the cancer ecosystem at multiple scales. Multiple molecular components such as integrin and cadherin and pathways (e.g., Rho GTPase), which are responsible for the cell-cell communications and the cell-environment interactions, have been revealed (Brücher and Jamall, 2014). They represent potential drug targets to perturb cancer cell-cell interactions and to constraint tumor growth. The Cancer Genome Atlas has identified millions of somatic mutations (Ledford, 2015). Correlated with generic variations, drug response phenomics data are available at molecular, cellular, tissue, and organism levels (Zbuk and Eng, 2007). The systematic integration of these data may allow us to predict drug-response phenotypes for intervention against multiple targets and pathways. Single cell sequencing has revealed the clonal evolution of breast cancer (Wang et al., 2014) and childhood acute lymphoblastic leukemia (Gawad et al., 2014), and drug resistance dynamics (Lee et al., 2014). These studies will provide critical information to predict the cancer evolutionary trajectory, leading to the development of pre-emptive treatment strategies that are in contrast to current reactive clinical approaches. In spite of this progress, one of fundamental challenges remaining is to bridge genetic and molecular mechanisms of single cell-cell interactions to the ecological dynamics of the cancer population.

Multi-scale modeling and simulation may play a key role in predicting the evolutionary dynamics of the cancer ecosystem, and identify anti-cancer therapeutic targets for pre-emptive treatment. It is possible to reconstruct context-specific whole cell models by integrating multiple omics data (Karr et al., 2012). Subsequently, their cellular functions can be simulated and predicted at different evolutionary stages under a framework of constraint-based modeling (Bordbar et al., 2014). Using a single cell or sub-clone as the building block, the cancer ecosystem can be modeled as a dynamic cell-cell interaction network, in which the node is a cell, and the edge represents the cell-cell interaction. Each node, or cluster of nodes, has different traits and evolutionary trajectory, yet depend on each other, as shown in Figure 1. A network representation may allow us to understand the emergent properties of the cancer ecosystem. For example, one of intrinsic properties of biological network is “robust-yet-fragile” (Kitano, 2007). The removal of a single node may have little impact on the whole system. However, the weak perturbation of multiple nodes can lead to the system failure, even if these nodes are not deleted. A number of effective therapies in treating complex diseases may follow this principle (Xie et al., 2012). For instance, successful anti-psychotic drugs, such as clozapine, mediate their effects through binding entire families of serotonin and dopamine receptors. The clinical failures of many anti-psychotic drugs can be attributed to them being too selective as designed (Hopkins et al., 2006). In another example, the anti-cancer effect of HIV protease inhibitors is proposed to comes from their weak bindings to multiple kinases (Xie et al., 2011). Moreover, the perturbation of edges may be more effective than nodes to regulate the state transition of a non-linear dynamic system (Tong et al., 2012). An additional advantage of edge perturbation is that the cancer cell has little selection pressure to evolve into a drug resistant phenotype, as the cancer cell will not be killed directly by the drug. In a proof-of-concept study, blocking cell-cell communication inhibited the repopulation of cancer stem cells, thus enhancing the effectiveness of anti-cancer therapy (Kurtova et al., 2015). To capture the whole dynamic spectrum of the cancer ecosystem, mechanistic and quantitative dynamic simulation is needed. Coarse-grained dynamic modeling has successfully identified an optimized sequence of therapies to improve the survival of patients with metastatic castrate resistant prostate cancer (Gallaher et al., 2014). Agent-based modeling that has been successfully applied to study the dynamics of complex systems could be a powerful tool to integrate whole cell models into a dynamic model of the cancer ecosystem (An et al., 2009).

**Figure 1:**
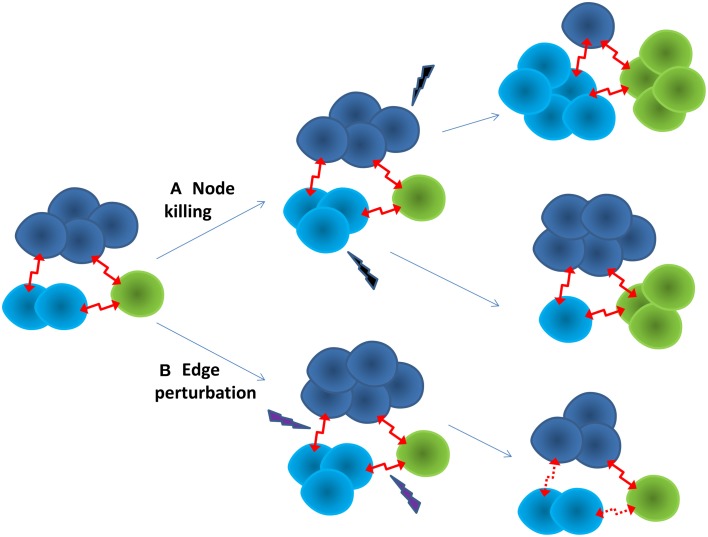
**Two strategies for anti-cancer therapy**. **(A)** Node killing strategy kills sub-clones of cancer cells through chemo-, targeted-, and immuno-therapy. The adaptive evolution of the cancer often leads to drug resistance. The cancer often turns into a more aggressive form. **(B)** Edge perturbation strategy aims to disturb the cell-cell interactions of the cancer ecosystem. It may have bigger impact on the cancer as a whole. It is less likely for the cancer to evolve into a drug resistance phenotype, as no sub-clone can gain particular evolutionary advantage.

Multi-target therapy can be achieved by either polypharmacology or drug combination. Polypharmacology has several advantages over drug combination. Firstly, it is not a trivial task to optimize dosages and sequences of a drug combination. The highly heterogeneous nature of cancer cells makes optimization even more difficult. Secondly, potential drug-drug interactions may cause serious side-effects. A singe “dirty” drug may reduce the probability of this problem. Finally, it is argued that polypharmacology is more likely to achieve desired selective profiles than the drug combination (Varshavsky, 1998). Compared with anti-virulence agents for bacteria, selectivity is particularly challenging in the development of anti-cancer therapy, as the normal cell is more similar to the cancer cell than the bacteria. The side-effect of anti-cancer therapy is mainly because the drug cannot distinguish the normal cell from the cancer cell. The cancer genes that harbor driver mutations can be either up-regulated or down-regulated. A polypharmacological agent can be designed in such a way that it is *mutually exclusive*—it binds both the up-regulated gene A and the down-regulated gene B (terms A+ and B-, respectively) (Varshavsky, 1998). Consequently, the agent will selectively bind to A+ in the cancer cell as B- is less competitive. In the normal cell, A and B will competitively bind the agent, thus the agent will have little impact on the function of either of them. In contrast, the combination of two drugs, which bind to A and B, respectively, will be less selective. Following the same principle above, a selective agent can be designed for genes that are all up-regulated through targeting the allosteric site of one gene (Varshavsky, 1998).

The identification of suitable therapeutic targets is only the starting point of anti-cancer drug development. It is often more challenging to discover molecules that are able to achieve desirable therapeutic effects with minimum side effects. In addition to conventional druggable targets, targeting the protein-protein interaction (PPI) interface could be an efficient strategy to modulate signal transduction, cell-cell communications, and cell-environmental interactions (Jubb et al., 2012; Mullard, 2012). For example, cadherin-p120 interactions may mediate the contact inhibition of locomotion (CIL) that controls cell growth. The loss of CIL leads to tumor growth and metastasis (Mayor and Carmona-Fontaine, 2010). Thus, enhancing cancer cell-cell interactions may inhibit tumor progression. Historically, it is difficult to design small molecule inhibitors to target the PPI interface, as it is flat, large, and featureless, and thus considered undruggable. Multiple weak binders instead of a single strong binder could be an alternative strategy to designing PPI modulators, as the PPI interface is characterized by “hot spots” that contribute the most to the binding free energy, and well-defined grooves or small pockets that are associated with a continuous epitope binding partner (Ma and Nussinov, 2014). As mentioned previously, multiple weak binders may be more effective in regulating biological systems than a single selective inhibitor with high binding affinity. Although drug combinations are a successful multi-target therapy strategy, possible drug-drug interactions may limit the number of drugs administrated together. Polypharmacology offers two alternatives to the drug combination. Polypharmacology aims to design “dirty” drugs that can bind to multiple receptors simultaneously. Its effectiveness in treating systematic diseases has been documented (Xie et al., 2012). For example, targeted polypharmacological agents have been successfully designed to modulate signaling transduction events (Apsel et al., 2008). It is possible for a small molecule to target multiple PPI interfaces, as they are promiscuous across fold space (Gao and Skolnick, 2010), and may share conserved hot spot residues (Keskin et al., 2005; Shulman-Peleg et al., 2007). The desired multi-target binding specificity can be achieved through the fine-tuned side chain geometry and chemistry of protein-ligand interactions (Gao and Skolnick, 2010).

In summary, with advances in whole genome sequencing, high-throughput techniques, systems biology, and cloud computing, information on the evolutionary dynamics of cancer, from a single cell to the whole population, is starting to emerge. Concurrently, progress in medicinal chemistry is expanding the druggable target space (e.g., through targeting the protein-protein interaction interface and allosteric events). Putting these efforts together may open a new door to multi-target therapies for cancer treatment.

## Conflict of interest statement

The authors declare that the research was conducted in the absence of any commercial or financial relationships that could be construed as a potential conflict of interest.
